# Postoperative Complication and Reoperation Rates Following Resection of Soft Tissue vs. Bone Malignancies Based on Anatomic Location in the Inpatient Setting

**DOI:** 10.1155/2023/5455719

**Published:** 2023-03-10

**Authors:** Alexander M. Ballatori, Shane Shahrestani, Andy Ton, Xiao T. Chen, Tarek Yamout, Brandon S. Gettleman, Nathanael D. Heckmann, Lawrence R. Menendez, Alexander B. Christ

**Affiliations:** ^1^Department of Orthopaedic Surgery, Keck School of Medicine of USC, Los Angeles, California, USA; ^2^Virginia Spine Institute, Reston, Virginia, USA; ^3^University of South Carolina School of Medicine, Columbia, SC, USA

## Abstract

**Introduction:**

Surgical excisions of upper and lower extremity malignancies are increasing annually, due in part to the rising incidence of sarcomas. The purpose of this study is to compare readmissions, reoperation rate, and complications following surgical excision of soft/connective tissue vs bone malignancies of the upper and lower extremities.

**Methods:**

The Nationwide Readmissions Database (NRD) was queried from 2016-2017 to conduct a retrospective analysis of 16,435 patients diagnosed with malignant neoplasms of the long bone (ULLB, *n* = 1,433) and soft tissue (ULST, *n* = 2,049) of the upper limb and malignant neoplasms of the long bone (LLLB, *n* = 5,422) and soft tissue (LLST, *n* = 7,531) of the lower limb. Patients who underwent surgical excision of their neoplasms were included. Binomial multivariate logistic regression was used to compare complications, nonelective readmission rates, and reoperation rates between the two groups at 30 and 90 days.

**Results:**

Average age of the ULST group was 61.88, with 36% female. Average age of the ULLB group was 44.97, with 41.90% female. Average age of the LLST group was 60.96, with 46.90% female. Average age of the LLLB group was 43.09, with 42.60% female. The ULST group had lower odds of readmission within 30 days (*p*=0.263), which became significant within 90 days of surgery (*p*=0.045). The LLST group had significantly higher odds of infection, reoperation within 30 to 90 days of the index surgery compared to the LLLB group (*p* < 0.0001). The LLST group had significantly lower odds of readmission within 30 (*p*=0.04) and 90 days (*p*=0.015) of the index surgery.

**Conclusion:**

Patients in the ULST group had significantly lower odds of 90-day readmission compared to the ULLB group. There were also significantly lower odds of 30- and 90-day readmission in the LLST group compared to the LLLB group. However, the LLST group had significantly higher odds of infection and reoperation within 30 and 90 days compared to the LLLB group.

## 1. Introduction

Surgical resections of upper and lower extremity malignancies are increasing annually within the United States, due in part to the rising incidence of sarcomas [1]. Numerous subtypes of sarcoma exist, and definitive guidelines for management for every subtype do not yet exist due to the rarity and heterogeneity of these malignancies. The vast majority require surgical resection for cure; however, it is difficult to draw conclusions regarding complication profiles for each type of resection due to variation in location, histology, size, and host factors. Further research is required to better understand complication rates amongst different types of sarcoma resections in the extremities [2–5].

Malignancy and surgery are both independent risk factors for thromboembolic events, and prophylactic anticoagulation is recommended for most sarcoma resections [6]. The rates of thromboembolic events based on whether the malignancy is of the soft/connective tissue or bone origin is yet to be studied. Therefore, the appropriate thromboembolic management concerning surgical removal of upper and lower limb sarcomas is still currently under investigation. It is unclear whether upper extremity procedures pose a similar thromboembolic risk as lower extremity procedures, and whether there is a difference in the rate of postoperative thromboembolism between bone and soft tissue resections [7, 8].

The literature is limited with respect to understanding the differences in surgical management between the soft/connective tissue and bone sarcomas due to the heterogeneity of these diseases. In addition, early postoperative outcomes following removal of upper and lower limb malignant neoplasms from bone versus soft tissue have not been compared. Further understanding of these risks can help guide clinical decision-making with respect to prophylactic measures for patients who are predisposed to certain postoperative outcomes. Therefore, the purpose of this study is to compare readmissions, reoperation rate, and complications following surgical resection of soft/connective tissue versus bone malignancies of the upper and lower limbs.

## 2. Methods

### 2.1. Data Source

The data source utilized in this study is the Healthcare Cost and Utilization Project (HCUP) Nationwide Readmissions Database (NRD) from 2016 to 2017. The NRD is a yearly nationally representative inpatient database from the Agency for Healthcare Research and Quality (AHRQ) with information regarding patient demographics, diagnoses, procedures, and readmissions. Patients are deidentified and are each represented as unique patient linkages to allow for accurate patient tracking throughout the calendar year. The NRD is publicly available for purchase and has been designed to allow for the nationally representative readmission analysis. Between NRD 2016 and 2017, we identified more than 35 million patient discharges, and all data regarding patient diagnoses and procedures were queried using International Classification of Diseases, Tenth Revision (ICD-10) codes in all patient admissions and readmissions. NRD years 2015 and earlier were excluded due to their usage of ICD-9 coding, which lacked the granularity in the procedure type and postoperative complications necessary for the analysis. Charges described in this study reflect total hospital charges not including professional fees and noncovered charges. Institutional Review Board (IRB) approval and informed consent were not required as we used a deidentified publicly available database.

### 2.2. Patient Selection Criteria

We conducted a retrospective cohort analysis of 6,855 patients with malignant neoplasms of long bones (upper limb: 1,433; lower limb: 5,422) and 9,580 patients with malignant neoplasms of connective/soft tissues (upper limb: 2,049; lower limb: 7,531) based on ICD-10 coding (Supplementary Table 1). From these groups, we further subdivided those who received a surgical procedure to remove their neoplasms, and compared complications, readmission rates, and reoperation rates by the anatomical region; upper limb soft/connective tissue (ULST), upper limb long bone (ULLB), lower limb soft/connective tissue (LLST), and lower limb long bone (LLLB). Complications were queried and compared through multivariate statistics.

### 2.3. Multivariate Analysis

Binomial multivariate logistic regression was used to compare patients who underwent resection of a malignant neoplasm of bone versus connective/soft tissue in the upper and lower limbs. This method was used due to variation in age, sex, and CCI between the two groups. Dependent variables within 30 days included DVT, PE, infection, readmission, and reoperation rates (Supplementary Table 2). Dependent variables within 90 days included readmission and reoperation rates. Independent covariates were age, sex, and CCI. Wald testing was performed to evaluate the effect of the weighted distance between the estimated value and the hypothesized true value under the null hypothesis on statistical parameters within each model. All statistics were conducted in RStudio (Version 1.2.5042) with *α* = 0.05 level of significance.

## 3. Results

### 3.1. Demographics

We identified 692 patients with ULST malignant neoplasms and 272 patients with ULLB malignant neoplasms who received surgical resection of their malignancies. The average age of the ULST group was 61.88 ± 20.05, with 36% female, and an average CCI of 3.14 ± 1.76. The average age of the ULLB group was 44.97 ± 21.87, with 41.90% female, and an average CCI of 1.98 ± 1.92 (Table 1).

We identified 3,195 patients with LLST malignant neoplasms and 1,089 patients with LLLB malignant neoplasms who received surgical resection of their malignancies. The average age of the LLST group was 60.96 ± 17.93, with 46.90% female, and an average CCI of 3.04 ± 1.74. The average age of the LLLB group was 43.09 ± 25.28, with 42.60% female, and an average CCI of 1.89 ± 1.96 (Table 1).

### 3.2. Primary Stay

The ULST group had a longer average LOS compared to the ULLB group (ULST: 5.36 ± 9.09 days; ULLB 4.35 ± 5.4). However, the ULLB group had higher total costs from their primary admission (ULLB: $27,637.11 ± $20,610.53; ULST: $19,112.26 ± 23,733.97). In terms of complications, the ULST group had higher rates of infection, but lower rates of DVT and PE at the primary stay compared to the ULLB group (Table 2).

The LLLB group had a longer average LOS compared to the LLST (LLLB: 7.87 ± 8.61 days; LLST: 5.69 ± 7.54). In addition, the LLLB group had higher total costs from their primary admission (LLLB: $36,883.72 ± $34,407.58; LLST: $19,974.00 ± $20,349.39). In terms of complications, the LLST group had higher rates of infections compared to the LLLB group at the primary stay (LLST: 7.32%; LLLB: 3.66%). The LLST group had higher rates of DVT and lower rates of PE compared to the LLLB group at the primary stay (Table 2).

### 3.3. Multivariate Analysis

After controlling for age, sex, and CCI, our multivariate analysis found that the ULST group had higher odds of DVT (OR: 6.3; 95% CI: 0.06–165,667; *p*=0.532) and infection (OR: 2.87; 95% CI: 0.601–17.3; *p*=0.209) compared to the ULLB group. Regarding readmission and reoperation rates, the ULST group had higher odds of reoperation within 30 (OR: 3.07; 95% CI: 0.672–18.0; *p*=0.172) and 90 days (OR: 1.74; 95% CI: 0.529–6.48; *p*=0.378)(Table 3). However, the ULST group had lower odds of readmission within 30 days (OR: 0.722; 95% CI: 0.410–1.29; *p*=0.263), which became significant within 90 days of surgery (OR: 0.593; 95% CI: 0.357–0.992; *p*=0.045) (Table 4).

After controlling for age, sex, and CCI, our multivariate analysis found that the LLST group had higher odds of DVT (OR: 1.98; 95% CI: 0.737–6.42; *p*=0.21) and lower odds of PE (OR: 0.407; 95% CI: 0.101–1.85; *p* = 0.215) compared to the LLLB group. In addition, the LLST group had significantly higher odds of infection (OR: 3.29; 95% CI: 1.88–5.99; *p* < 0.0001), reoperation within 30 (OR: 8.74; 95% CI: 3.86–23.7; *p* < 0.0001) and 90 days of the index surgery (OR: 7.45; 95% CI: 3.84–16.0; *p* < 0.0001) compared to the LLLB group. However, the LLST group had significantly lower odds of readmission within 30 (OR: 0.768; 95% CI: 0.598–0.990; *p*=0.04) and 90 days of the index surgery (OR: 0.738; 95% CI: 0.577–0.944; *p*=0.015) (Table 5).

## 4. Discussion

After controlling for age, sex, and CCI, the present study found significantly lower odds of 90-day readmission in the ULST group compared to the ULLB group, as well as significantly lower odds of 30- and 90-day readmission in the LLST group compared to the LLLB group. However, the LLST group had significantly higher odds of infection and reoperation within 30 and 90 days compared to the LLLB group.

Resection of the lower extremity soft tissue sarcoma having a higher complication profile than the upper extremity soft tissue sarcoma is a clinical outcome that has been established previously in the literature [9]. Our study sought to further analyze these isolated differences between the anatomic location and type of malignancy resected through utilization of a national database. The present study did not evaluate the long-term outcomes for our patients, as the NRD only allows patients to be tracked within the set calendar year. However, the immediate and short-term postoperative outcomes are necessary to understand when managing patients with complicated diseases.

Definitive treatment of the soft tissue sarcoma involves wide resection of the tumor with adequate margins, with the secondary goal of preserving the complicated musculoskeletal functions of the upper limb [7, 10]. Our study suggests that following these complex procedures, the rates of DVT and infection are higher in the soft tissue group than the long bone sarcomas. Although we report differences in DVT and infection in the upper limb, these findings were not significant. The only significant difference found between the two groups was with respect to lower odds of 90-day readmissions in the ULST sarcoma group.

We theorize that the higher, but not significant, rate of increased infection in the ULST is due to the complex soft tissue coverage required in these cases, as well as the need for radiation therapy in cases of high-grade sarcoma or close margin resection. Flap reconstruction is an essential component of UE STS surgery, and in many cases may require several operations to achieve definitive healing [11, 12]. The higher odds of 90-day readmissions in ULLB may be related to mechanical complications, as these resections require reconstruction with a variety of joint replacing and preserving techniques, many of which carry significant complication rates [13–16].

As for lower extremity sarcomas, a recent meta-analysis of lower extremity limb-salvage surgery for sarcoma found that current techniques have improved limb-salvage rates, but not without substantial postoperative complications [17–19]. Our study found infection rates of 7.32% and 3.66% and DVT rates of 1.90% and 1.10% at the primary stay for LLST and LLLB sarcomas, respectively. In addition, we found that 9.57% of LLST and 2.76% of LLLB sarcomas required reoperation within 90 days of their index admission. When we compared the differences between LLST and LLLB in our multivariate model, we found that the LLST group had significantly higher odds of infection and reoperation within 30 and 90 days than the LLLB group after controlling for age, sex, and CCI.

It is important to note that findings in the current study were subject to several limitations. In using a national registry, our study was subject to limitations inherent to ICD-9 and ICD-10 coding. For example, since this is an inpatient source in which numerous are resected and discharged as outpatients, we are unable to capture a certain subset of patients. However, given the sample size over a two-year period, the number of tumors we see are consistent with known epidemiologic incidence of these types of tumors in both the upper and lower extremity leading us to believe these results are accurate and valuable for clinics to consider when treating their patients. The dichotomous nature of these diagnoses and procedure codes limits further granularity in characterizing diagnosis severity or operative details. For instance, our study observed a higher postoperative infection rate across soft tissue sarcomas in both the upper and lower extremities. As prior studies have established, this was most likely owed to a history of radiation therapy amongst the sarcoma group, thereby predisposing them to higher infection risks [20, 21]. We were, however, unable to confirm this association within our cohort, given the limited detail into patients' treatment history offered by the NRD and ICD coding.

Despite the limited granularity permitted by use of ICD coding, we were able to include a large, nationally representative sample to achieve a highly powered analysis. The concordance of findings between our study and the literature also substantiates the validity of the database, lending to the feasibility of future studies comparing epidemiological trends and treatment outcomes in parallel to new surgical developments for malignant soft tissue and bone sarcomas. Moreover, given that the NRD is an inpatient database, this study is comprised of patients who are operatively managed and require inpatient admission. This population likely represents a higher disease burden considering that an appreciable proportion of patients who undergo surgical resection of soft tissue sarcomas are discharged the same day as outpatients.

## 5. Conclusion

Bone and soft tissue sarcomas are still challenging clinical entities and require a multidisciplinary approach for the best management [22]. This study highlights, on a national scale, the distinctive postoperative outcomes based on the location and type of sarcoma a patient has. In conclusion, future research is required to fully characterize the unique postoperative complications and long-term outcomes following resection of these rare malignancies.

## Figures and Tables

**Table 1:** Demographics.

	Resection of malignant neoplasm, demographics			
	Upper limb		Lower limb	
	Soft tissue	Long bone	Soft tissue	Long bone
*n*	692	272	3,195	1,089
Age (SD)	61.88 (20.05)	44.97 (21.87)	60.96 (17.93)	43.09 (25.28)
Female	36%	41.90%	46.90%	42.60%
CCI (SD)	3.14 (1.76)	1.98 (1.92)	3.04 (1.74)	1.89 (1.96)

**Table 2:** Overview of primary admission.

	Resection of malignant neoplasm, primary stay			
	Upper limb		Lower limb	
	Soft tissue	Long bone	Soft tissue	Long bone
LOS (SD)	5.36 (9.09)	4.35 (5.4)	5.69 (7.54)	7.87 (8.61)
Cost (SD)	$19, 112.26 ($23, 733.97)	$27, 637.11 ($20, 610.53)	$19, 974 ($20, 349.39)	$36, 883.72 ($34, 407.58)
Infection	2.71%	2.09%	7.32%	3.66%
DVT	0.25%	0.55%	1.90%	1.10%
PE	0%	0.57%	0.28%	0.96%

**Table 3:** Readmission and reoperation rates at 30 and 90 days following the primary admission.

	Resection of malignant neoplasm, readmission/reoperation rates			
	Upper limb		Lower limb	
	Soft tissue (%)	Long bone (%)	Soft tissue (%)	Long bone (%)
Readmission rate, 30 day	13.24	23.43	18.73	34.93
Reoperation rate, 30 day	3.86	1.78	5.53	1.20
Readmission rate, 90 day	21.01	35.82	29.93	47.54
Reoperation rate, 90 day	4.73	3.35	9.57	2.76

**Table 4:** Odds of complications, readmissions, and reoperations following resection of malignant neoplasms from the upper limb.

	Resection of malignant neoplasm, upper limb		
	OR	95% CI	*p* value
DVT	6.3	0.06–165,667	0.532
PE	NEO	NEO	NEO
Infection	2.87	0.601–17.3	0.209
Readmission rate, 30 day	0.722	0.410–1.29	0.263
Reoperation rate, 30 day	3.07	0.672–18.0	0.172
Readmission rate, 90 day	0.593	0.357–0.992	0.045
Reoperation rate, 90 day	1.74	0.529–6.48	0.378

OR >1 indicates higher odds in the soft/connective tissue group. NEO = not enough observations.

**Table 5:** Odds of complications, readmissions, and reoperations following resection of malignant neoplasms from the lower limb.

	Resection of malignant neoplasm, lower limb		
	OR	95% CI	*p* value
DVT	1.98	0.737–6.42	0.21
PE	0.407	0.101–1.85	0.215
Infection	3.29	1.88–5.99	<0.0001
Readmission rate, 30 day	0.768	0.598–0.990	0.04
Reoperation rate, 30 day	8.74	3.86–23.7	<0.0001
Readmission rate, 90 day	0.738	0.577–0.944	0.015
Reoperation rate, 90 day	7.45	3.84–16.0	<0.0001

OR > 1 indicates higher odds in the soft/connective tissue group.

## Data Availability

The data source (Healthcare Cost and Utilization Project Nationwide Readmissions Database) is deidentified, and therefore this study was exempted from Institutional Review Board approval.

## Supplementary Materials

Supplementary Materials Supplementary Table 1. ICD-10 diagnosis codes and procedure codes. Supplementary Table 2. ICD-10 diagnosis codes for complications.
