# Supplementary material for: Postoperative Complication and Reoperation Rates Following Resection of Soft Tissue vs. Bone Malignancies Based on Anatomic Location in the Inpatient Setting
Source: Sarcoma. 2023 Mar 10;2023:5455719. doi: 10.1155/2023/5455719 (PMC10023224; doi:10.1155/2023/5455719)
Supplement: Supplementary Materials — Supplementary Table 1. ICD-10 diagnosis codes and procedure codes. Supplementary Table 2. ICD-10 diagnosis codes for complications. [file 5455719.f1.zip › Supplemental Table 1 (1).docx]

| **Supplemental Table 1.** ICD-10 diagnosis and procedure codes. | | | |
| --- | --- | --- | --- |
| Description | | | ICD-10 Codes |
| Upper Limb | Long Bone | Malignant Neoplasm of Long Bone, Upper Limb | “C4000”, “C4001”, “C4002” |
|  |  | Excision, Scapula | “0PB50ZX”, “0PB50ZZ”, “0PB53ZX”, “0PB53ZZ”, “0PB54ZX”, “0PB54ZZ”, “0PB60ZX”, “0PB60ZZ”, “0PB63ZX”, “0PB63ZZ”, “0PB64ZX”, “0PB64ZZ” |
|  |  | Excision, Glenoid Cavity | “0PB70ZX”, “0PB70ZZ”, “0PB73ZX”, “0PB73ZZ”, “0PB74ZX”, “0PB74ZZ”, “0PB80ZX”, “0PB80ZZ”, “0PB83ZX”, “0PB83ZZ”, “0PB84ZX”, “0PB84ZZ” |
|  |  | Excision, Clavicle | “0PB90ZX”, “0PB90ZZ”, “0PB93ZX”, “0PB93ZZ”, “0PB94ZX”, “0PB94ZZ”,“0PBB0ZX”, “0PBB0ZZ”, “0PBB3ZX”, “0PBB3ZZ”, “0PBB4ZX”, “0PBB4ZZ” |
|  |  | Excision, Humeral Head | “0PBC0ZX”, “0PBC0ZZ”, “0PBC3ZX”, “0PBC3ZZ”, “0PBC4ZX”, “0PBC4ZZ”, “0PBD0ZX”, “0PBD0ZZ”, “0PBD3ZX”, “0PBD3ZZ”, “0PBD4ZX”, “0PBD4ZZ” |
|  |  | Excision, Humeral Shaft | “0PBF0ZX”, “0PBF0ZZ”, “0PBF3ZX”, “0PBF3ZZ”, “0PBF4ZX”, “0PBF4ZZ”, “0PBG0ZX”, “0PBG0ZZ”, “0PBG3ZX”, “0PBG3ZZ”, “0PBG4ZX”, “0PBG4ZZ” |
|  |  | Excision, Radius | “0PBH0ZX”, “0PBH0ZZ”, “0PBH3ZX”, “0PBH3ZZ”, “0PBH4ZX”, “0PBH4ZZ”, “0PBJ0ZX”, “0PBJ0ZZ”, “0PBJ3ZX”, “0PBJ3ZZ”, “0PBJ4ZX”, “0PBJ4ZZ” |
|  |  | Excision, Ulna | “0PBK0ZX”, “0PBK0ZZ”, “0PBK3ZX”, “0PBK3ZZ”, “0PBK4ZX”, “0PBK4ZZ”, “0PBL0ZX”, “0PBL0ZZ”, “0PBL3ZX”, “0PBL3ZZ”, “0PBL4ZX”, “0PBL4ZZ” |
|  |  | Excision, Carpal | “0PBM0ZX”, “0PBM0ZZ”, “0PBM3ZX”, “0PBM3ZZ”, “0PBM4ZX”, “0PBM4ZZ”, “0PBN0ZX”, “0PBN0ZZ”, “0PBN3ZX”, “0PBN3ZZ”, “0PBN4ZX”, “0PBN4ZZ” |
|  |  | Excision, Metacarpal | “0PBP0ZX”, “0PBP0ZZ”, “0PBP3ZX”, “0PBP3ZZ”, “0PBP4ZX”, “0PBP4ZZ”, “0PBQ0ZX”, “0PBQ0ZZ”, “0PBQ3ZX”, “0PBQ3ZZ”, “0PBQ4ZX”, “0PBQ4ZZ” |
|  |  | Excision, Thumb Phalanx | “0PBR0ZX”, “0PBR0ZZ”, “0PBR3ZX”, “0PBR3ZZ”, “0PBR4ZX”, “0PBR4ZZ”, “0PBS0ZX”, “0PBS0ZZ”, “0PBS3ZX”, “0PBS3ZZ”, “0PBS4ZX”, “0PBS4ZZ” |
|  |  | Excision, Finder Phalanx | “0PBT0ZX”, “0PBT0ZZ”, “0PBT3ZX”, “0PBT3ZZ”, “0PBT4ZX”, “0PBT4ZZ”, “0PBV0ZX”, “0PBV0ZZ”, “0PBV3ZX”, “0PBV3ZZ”, “0PBV4ZX”, “0PBV4ZZ” |
|  | Connective and Soft Tissue | Malignant Neoplasm of Connective and Soft Tissue of Upper Limb, Including Shoulder | “C4910”, “C4911”, “C4912” |
|  |  | Excision, Upper Arteries | “03B50ZX”, “03B50ZZ”, “03B53ZX”, “03B53ZZ”, “03B54ZX”, “03B54ZZ”, “03B60ZX”, “03B60ZZ”, “03B63ZX”, “03B63ZZ”, “03B64ZX”, “03B64ZZ”, “03B70ZX”, “03B70ZZ”, “03B73ZX”, “03B73ZZ”, “03B74ZX”, “03B74ZZ”, “03B80ZX”, “03B80ZZ”, “03B83ZX”, “03B83ZZ”, “03B84ZX”, “03B84ZZ”, “03B90ZX”, “03B90ZZ”, “03B93ZX”, “03B93ZZ”, “03B94ZX”, “03B94ZZ”, “03BA0ZX”, “03BA0ZZ”, “03BA3ZX”, “03BA3ZZ”, “03BA4ZX”, “03BA4ZZ”, “03BB0ZX”, “03BB0ZZ”, “03BB3ZX”, “03BB3ZZ”, “03BB4ZX”, “03BB4ZZ”, “03BC0ZX”, “03BC0ZZ”, “03BC3ZX”, “03BC3ZZ”, “03BC4ZX”, “03BC4ZZ”, “03BD0ZX”, “03BD0ZZ”, “03BD3ZX”, “03BD3ZZ”, “03BD4ZX”, “03BD4ZZ”, “03BF0ZX”, “03BF0ZZ”, “03BF3ZX”, “03BF3ZZ”, “03BF4ZX”, “03BF4ZZ” |
|  |  | Excision, Upper Veins | “05B70ZX”, “05B70ZZ”, “05B73ZX”, “05B73ZZ”, “05B74ZX”, “05B74ZZ”, “05B80ZX”, “05B80ZZ”, “05B83ZX”, “05B83ZZ”, “05B84ZX”, “05B84ZZ”, “05B90ZX”, “05B90ZZ”, “05B93ZX”, “05B93ZZ”, “05B94ZX”, “05B94ZZ”, “05BA0ZX”, “05BA0ZZ”, “05BA3ZX”, “05BA3ZZ”, “05BA4ZX”, “05BA4ZZ”, “05BB0ZX”, “05BB0ZZ”, “05BB3ZX”, “05BB3ZZ”, “05BB4ZX”, “05BB4ZZ”, “05BC0ZX”, “05BC0ZZ”, “05BC3ZX”, “05BC3ZZ”, “05BC4ZX”, “05BC4ZZ”, “05BD0ZX”, “05BD0ZZ”, “05BD3ZX”, “05BD3ZZ”, “05BD4ZX”, “05BD4ZZ”, “05BF0ZX”, “05BF0ZZ”, “05BF3ZX”, “05BF3ZZ”, “05BF4ZX”, “05BF4ZZ”, “05BG0ZX”, “05BG0ZZ”, “05BG3ZX”, “05BG3ZZ”, “05BG4ZX”, “05BG4ZZ”, “05BH0ZX”, “05BH0ZZ”, “05BH3ZX”, “05BH3ZZ”, “05BH4ZX”, “05BH4ZZ” |
|  |  | Excision, Lymphatic and Hemic Systems | “07B30ZX”, “07B30ZZ”, “07B33ZX”, “07B33ZZ”, “07B34ZX”, “07B34ZZ”, “07B40ZX”, “07B40ZZ”, “07B43ZX”, “07B43ZZ”, “07B44ZX”, “07B44ZZ”, “07B50ZX”, “07B50ZZ”, “07B53ZX”, “07B53ZZ”, “07B54ZX”, “07B54ZZ”, “07B60ZX”, “07B60ZZ”, “07B63ZX”, “07B63ZZ”, “07B64ZX”, “07B64ZZ”, “07BC0ZX”, “07BC0ZZ”, “07BC3ZX”, “07BC3ZZ”, “07BC4ZX”, “07BC4ZZ” |
|  |  | Excision, Skin | “0HBBXZX”, “0HBBXZZ”, “0HBCXZX”, “0HBCXZZ”, “0HBDXZX”, “0HBDXZZ”, “0HBEXZX”, “0HBEXZZ”, “0HBFXZX”, “0HBFXZZ”, “0HBGXZX”, “0HBGXZZ”, “0HBQXZX”, “0HBQXZZ” |
|  |  | Excision, Subcuataneous Tissue and Fascia | “0JBD0ZX”, “0JBD0ZZ”, “0JBD3ZX”, “0JBD3ZZ”, “0JBF0ZX”, “0JBF0ZZ”, “0JBF3ZX”, “0JBF3ZZ”, “0JBG0ZX”, “0JBG0ZZ”, “0JBG3ZX”, “0JBG3ZZ”, “0JBH0ZX”, “0JBH0ZZ”, “0JBH3ZX”, “0JBH3ZZ”, “0JBJ0ZX”, “0JBJ0ZZ”, “0JBJ3ZX”, “0JBJ3ZZ”, “0JBK0ZX”, “0JBK0ZZ”, “0JBK3ZX”, “0JBK3ZZ” |
|  |  | Excision, Muscle | “0KB50ZX”, “0KB50ZZ”, “0KB53ZX”, “0KB53ZZ”, “0KB54ZX”, “0KB54ZZ”, “0KB60ZX”, “0KB60ZZ”, “0KB63ZX”, “0KB63ZZ”, “0KB64ZX”, “0KB64ZZ”, “0KB70ZX”, “0KB70ZZ”, “0KB73ZX”, “0KB73ZZ”, “0KB74ZX”, “0KB74ZZ”, “0KB80ZX”, “0KB80ZZ”, “0KB83ZX”, “0KB83ZZ”, “0KB84ZX”, “0KB84ZZ”, “0KB90ZX”, “0KB90ZZ”, “0KB93ZX”, “0KB93ZZ”, “0KB94ZX”, “0KB94ZZ”, “0KBB0ZX”, “0KBB0ZZ”, “0KBB3ZX”, “0KBB3ZZ”, “0KBB4ZX”, “0KBB4ZZ”, “0KBC0ZX”, “0KBC0ZZ”, “0KBC3ZX”, “0KBC3ZZ”, “0KBC4ZX”, “0KBC4ZZ”, “0KBD0ZX”, “0KBD0ZZ”, “0KBD3ZX”, “0KBD3ZZ”, “0KBD4ZX”, “0KBD4ZZ” |
|  |  | Excision, Tendons | “0LB10ZX”, “0LB10ZZ”, “0LB13ZX”, “0LB13ZZ”, “0LB14ZX”, “0LB14ZZ”, “0LB20ZX”, “0LB20ZZ”, “0LB23ZX”, “0LB23ZZ”, “0LB24ZX”, “0LB24ZZ”, “0LB30ZX”, “0LB30ZZ”, “0LB33ZX”, “0LB33ZZ”, “0LB34ZX”, “0LB34ZZ”, “0LB40ZX”, “0LB40ZZ”, “0LB43ZX”, “0LB43ZZ”, “0LB44ZX”, “0LB44ZZ”, “0LB50ZX”, “0LB50ZZ”, “0LB53ZX”, “0LB53ZZ”, “0LB54ZX”, “0LB54ZZ”, “0LB60ZX”, “0LB60ZZ”, “0LB63ZX”, “0LB63ZZ”, “0LB64ZX”, “0LB64ZZ”, “0LB70ZX”, “0LB70ZZ”, “0LB73ZX”, “0LB73ZZ”, “0LB74ZX”, “0LB74ZZ”, “0LB80ZX”, “0LB80ZZ”, “0LB83ZX”, “0LB83ZZ”, “0LB84ZX”, “0LB84ZZ” |
|  |  | Excision, Bursae and Ligaments | “0MB10ZX”, “0MB10ZZ”, “0MB13ZX”, “0MB13ZZ”, “0MB14ZX”, “0MB14ZZ”, “0MB20ZX”, “0MB20ZZ”, “0MB23ZX”, “0MB23ZZ”, “0MB24ZX”, “0MB24ZZ”, “0MB30ZX”, “0MB30ZZ”, “0MB33ZX”, “0MB33ZZ”, “0MB34ZX”, “0MB34ZZ”, “0MB40ZX”, “0MB40ZZ”, “0MB43ZX”, “0MB43ZZ”, “0MB44ZX”, “0MB44ZZ”, “0MB50ZX”, “0MB50ZZ”, “0MB53ZX”, “0MB53ZZ”, “0MB54ZX”, “0MB54ZZ”, “0MB60ZX”, “0MB60ZZ”, “0MB63ZX”, “0MB63ZZ”, “0MB64ZX”, “0MB64ZZ”, “0MB70ZX”, “0MB70ZZ”, “0MB73ZX”, “0MB73ZZ”, “0MB74ZX”, “0MB74ZZ”, “0MB80ZX”, “0MB80ZZ”, “0MB83ZX”, “0MB83ZZ”, “0MB84ZX”, “0MB84ZZ”, “0MB90ZX”, “0MB90ZZ”, “0MB93ZX”, “0MB93ZZ”, “0MB94ZX”, “0MB94ZZ”, “0MBB0ZX”, “0MBB0ZZ”, “0MBB3ZX”, “0MBB3ZZ”, “0MBB4ZX”, “0MBB4ZZ” |
| Lower Limb | Long Bone | Malignant Neoplasm of Long Bone, Lower Limb | “C4020”, “C4021”, “C4022” |
|  |  | Excision, Pelvic Bone | “0QB20ZX”, “0QB20ZZ”, “0QB23ZX”, “0QB23ZZ”, “0QB24ZX”, “0QB24ZZ”, “0QB30ZX”, “0QB30ZZ”, “0QB33ZX”, “0QB33ZZ”, “0QB34ZX”, “0QB34ZZ” |
|  |  | Excision, Acetabulum | “0QB40ZX”, “0QB40ZZ”, “0QB43ZX”, “0QB43ZZ”, “0QB44ZX”, “0QB44ZZ”, “0QB50ZX”, “0QB50ZZ”, “0QB53ZX”, “0QB53ZZ”, “0QB54ZX”, “0QB54ZZ” |
|  |  | Excision, Upper Femur | “0QB60ZX”, “0QB60ZZ”, “0QB63ZX”, “0QB63ZZ”, “0QB64ZX”, “0QB64ZZ”, “0QB70ZX”, “0QB70ZZ”, “0QB73ZX”, “0QB73ZZ”, “0QB74ZX”, “0QB74ZZ” |
|  |  | Excision, Femoral Shaft | “0QB80ZX”, “0QB80ZZ”, “0QB83ZX”, “0QB83ZZ”, “0QB84ZX”, “0QB84ZZ”, “0QB90ZX”, “0QB90ZZ”, “0QB93ZX”, “0QB93ZZ”, “0QB94ZX”, “0QB94ZZ” |
|  |  | Excision, Lower Femur | “0QBB0ZX”, “0QBB0ZZ”, “0QBB3ZX”, “0QBB3ZZ”, “0QBB4ZX”, “0QBB4ZZ”, “0QBC0ZX”, “0QBC0ZZ”, “0QBC3ZX”, “0QBC3ZZ”, “0QBC4ZX”, “0QBC4ZZ” |
|  |  | Excision, Patella | “0QBD0ZX”, “0QBD0ZZ”, “0QBD3ZX”, “0QBD3ZZ”, “0QBD4ZX”, “0QBD4ZZ”, “0QBF0ZX”, “0QBF0ZZ”, “0QBF3ZX”, “0QBF3ZZ”, “0QBF4ZX”, “0QBF4ZZ” |
|  |  | Excision, Tibia | “0QBG0ZX”, “0QBG0ZZ”, “0QBG3ZX”, “0QBG3ZZ”, “0QBG4ZX”, “0QBG4ZZ”, “0QBH0ZX”, “0QBH0ZZ”, “0QBH3ZX”, “0QBH3ZZ”, “0QBH4ZX”, “0QBH4ZZ” |
|  |  | Excision, Fibula | “0QBJ0ZX”, “0QBJ0ZZ”, “0QBJ3ZX”, “0QBJ3ZZ”, “0QBJ4ZX”, “0QBJ4ZZ”, “0QBK0ZX”, “0QBK0ZZ”, “0QBK3ZX”, “0QBK3ZZ”, “0QBK4ZX”, “0QBK4ZZ” |
