# Supplementary material for: Postoperative Complication and Reoperation Rates Following Resection of Soft Tissue vs. Bone Malignancies Based on Anatomic Location in the Inpatient Setting
Source: Sarcoma. 2023 Mar 10;2023:5455719. doi: 10.1155/2023/5455719 (PMC10023224; doi:10.1155/2023/5455719)
Supplement: Supplementary Materials — Supplementary Table 1. ICD-10 diagnosis codes and procedure codes. Supplementary Table 2. ICD-10 diagnosis codes for complications. [file 5455719.f1.zip › Supplemental Table 2 (1).docx]

| **Supplemental Table 2.** ICD-10 diagnosis codes for complications. | | |
| --- | --- | --- |
| Description | | ICD-10 Codes |
| Complications | PE | “I2601”, “I2602”, “I2609”, “I2690”, “I2692”, “I2693”, “I2694”, “I2699” |
|  | DVT/Thromboembolic | “I82210”, “I82220”, “I82290”, “I823”, “I82401”, “I82402”, “I82403”, “I82409”, “I82411”, “I82412”, “I82413”, “I82419”, “I82421”, “I82422”, “I82423”, “I82429”, “I82431”, “I82432”, “I82433”, “I82439”, “I82441”, “I82442”, “I82443”, “I82449”, “I82451”, “I82452”, “I82453”, “I82459”, “I82461”, “I82462”, “I82463”, “I82469”, “I82491”, “I82492”, “I82493”, “I82499”, “I824Y1”, “I824Y2”, “I824Y3”, “I824Y9”, “I824Z1”, “I824Z2”, “I824Z3”, “I824Z9”, “I82601”, “I82602”, “I82603”, “I82609”, “I82611”, “I82612”, “I82613”, “I82619”, “I82621”, “I82622”, “I82623”, “I82629”, “I82A11”, “I82A12”, “I82A13”, “I82A19”, “I82B11”, “I82B12”, “I82B13”, “I82B19”, “I82C11”, “I82C12”, “I82C13”, “I82C19”, “I82811”, “I82812”, “I82813”, “I82819”, “I82890”, “I8290”, “I7410”, “I7411”, “I7419”, “I742”, “I743”, “I744”, “I745”, “I748”, “I749” |
|  | Infection | 'K6811', 'T814XXA', 'T8579XA', 'T80219A', 'T80211A', 'T80212A', 'T8022XA', 'T8029XA','T880XXA', 'A419', 'R6520', 'K6811', 'K6811', 'T8130XA', 'T8132XA', 'T8131XA', 'T8189XA','T8183XA', 'T8579XA', 'T8460XA', 'T847XXA', 'K6811', 'T8579XA', 'A419', 'R6520', 'T8130XA','T8132XA', 'T8131XA', 'T8133XA', 'T8183XA', 'T847XXA','T814XXA' |
